# Control of Cytokine Production by Human Fc Gamma Receptors: Implications for Pathogen Defense and Autoimmunity

**DOI:** 10.3389/fimmu.2015.00079

**Published:** 2015-02-24

**Authors:** Lisa T. C. Vogelpoel, Dominique L. P. Baeten, Esther C. de Jong, Jeroen den Dunnen

**Affiliations:** ^1^Department of Cell Biology and Histology, Academic Medical Center, University of Amsterdam, Amsterdam, Netherlands; ^2^Department of Clinical Immunology and Rheumatology, Academic Medical Center, University of Amsterdam, Amsterdam, Netherlands

**Keywords:** antibacterial response, cross-talk, dendritic cells, FcγRIIa, macrophages, rheumatoid arthritis, systemic lupus erythematosus, TNFα

## Abstract

Control of cytokine production by immune cells is pivotal for counteracting infections via orchestration of local and systemic inflammation. Although their contribution has long been underexposed, it has recently become clear that human Fc gamma receptors (FcγRs), which are receptors for the Fc region of immunoglobulin G (IgG) antibodies, play a critical role in this process by controlling tissue- and pathogen-specific cytokine production. Whereas individual stimulation of FcγRs does not evoke cytokine production, FcγRs cell-type specifically interact with various other receptors for selective amplification or inhibition of particular cytokines, thereby tailoring cytokine responses to the immunological context. The physiological function of FcγR-mediated control of cytokine production is to counteract infections with various classes of pathogens. Upon IgG opsonization, pathogens are simultaneously recognized by FcγRs as well as by various pathogen-sensing receptors, leading to the induction of pathogen class-specific immune responses. However, when erroneously activated, the same mechanism also contributes to the development of autoimmune diseases such as rheumatoid arthritis and systemic lupus erythematosus. In this review, we discuss control of cytokine production as a novel function of FcγRs in human innate immune cells in the context of homeostasis, infection, and autoimmunity and address the possibilities for future therapeutic exploitation.

## Introduction

Control of cytokine production is pivotal for controlling local and systemic inflammation and is required for shaping both innate and adaptive immune responses. Innate immune cells produce cytokines upon detection of pathogens or endogenous danger signals via activation of different families of receptors, which collectively are referred to as pattern recognition receptors (PRRs). The most well-known examples of PRRs are the families of toll-like receptors (TLRs), C-type lectin receptors, NOD-like receptors, and RIG-I-like receptors (1). However, the list of families of receptors that can induce or modulate cytokine production is still continuously expanding.

In the last 10 years, it has become apparent that also the family of Fc gamma receptors (FcγRs), which are receptors for the Fc region of immunoglobulin G (IgG) (2), plays a major role in orchestrating cytokine production. FcγRs have long been known to mediate a large variety of functions, such as antigen or pathogen uptake, degranulation, antigen presentation, and antibody-dependent cellular cytotoxicity (ADCC) [reviewed by Nimmerjahn and Ravetch (3) and Guilliams et al. (4)]. In contrast, their function in orchestrating inflammation by controlling the production of cytokines has long been underexposed. When evaluating recent findings, it appears that FcγR-mediated control of cytokine production is physiologically important to tailor immune responses to efficiently counteract pathogens. However, when activated undesirably, the same mechanism of FcγR-mediated cytokine induction is responsible for excessive inflammation as observed in autoimmune diseases that are associated with IgG autoantibodies, such as rheumatoid arthritis (RA) and systemic lupus erythematosus (SLE).

In general, most knowledge on FcγR biology comes from mouse studies. Although various FcγR functions are conserved between species, both IgG subclasses and FcγRs differ in a number of aspects between mouse and man (5). These differences impede translation of findings for particular FcγR features from mouse studies to the human situation and vice versa. Importantly, the capacity of FcγRs to induce or modulate cytokine production appears to substantially differ between species, as summarized in Box 1. This difference is likely to be caused by differential expression of FcγRIIa, which is the main cytokine-inducing receptor in humans, but has no direct homolog in mice (3–5). In this review, we will therefore mainly focus on data from studies using human cells or humanized mouse models and their relevance to understanding and potential treatment of human diseases.

Box 1 **FcγR-related differences between mouse and man.**

The orchestration of cytokine responses by activating low-affinity FcγRs clearly differs between humans and mice. Four key differences in this regard are summarized below.

- FcγRIIa, which is the main FcγR responsible for the induction of pro-inflammatory cytokine production by human cells, is not expressed in mice (3–5).
- In various human cell types, including dendritic cells (DCs) and macrophages, stimulation with immune complexes induces FcγR-dependent caspase-1 and inflammasome activation for the production of functional IL-β (6, 14, 45). In contrast, in mice, immune complexes inhibit inflammasome activation and IL-1β production (93).
- In humans, the cytokine profile induced by cross-talk between activating FcγRs and co-receptors is predominantly characterized by various pro-inflammatory cytokines, of which TNFα upregulation is most pronounced (6, 7, 14, 45, 59–61, 71, 72). In contrast, the cytokine profile induced by combined stimulation of murine DCs or macrophages with immune complexes and TLR ligands is characterized by elevated IL-10 and abrogated IL-12 production, whereas TNFα production is not affected or even reduced (14, 93–98).
- In humans, FcγR-TLR cross-talk results in enhanced Th17 responses (6, 14), while in mice, FcγR co-stimulation promotes Th2 responses (93, 96), which most likely results from the above mentioned differences in cytokine profiles by antigen-presenting cells.

### Context-dependent cytokine production by FcγRs

A key feature of FcγRs related to cytokine production is that FcγRs are unable to directly induce cytokines themselves, but instead collaborate with other receptors to amplify or inhibit the production of specific cytokines. The ultimate FcγR-mediated cytokine profile is therefore not uniform, but instead appears to be tailored to the immunological context in which FcγR stimulation takes place. We here propose that this context-dependent cytokine production mediated by FcγRs is achieved through regulation at (at least) four levels.

First, the induced cytokine profile depends on the specific receptor that FcγRs collaborate with. For example, cross-talk between FcγRIIa and TLRs, as occurs upon recognition of IgG opsonized bacteria, strongly amplifies production of pro-inflammatory cytokines such as TNFα (6). In contrast, FcγRs do not synergize with several cytokine receptors, including IL-6 receptor, IL-12 receptor, and IL-23 receptor (7). Second, the FcγR-mediated cytokine response depends on the balance of activating versus inhibitory FcγRs. Indeed, it has been shown that stimulation of human DCs with IgG immune complexes simultaneously conveys an inflammatory signal by triggering activating receptor FcγRIIa and a tolerogenic signal by triggering inhibitory receptor FcγRIIb (8). Disturbances of this balance between activating and inhibitory FcγRs are associated with inflammation as observed in patients with bacterial infections or RA (9–11). Third, FcγRs are able to discriminate between aggregated (i.e., antigen-bound) and soluble IgG, thereby adding another layer of complexity to FcγR-mediated cytokine modulation. For example, while large immune complexes are known to enhance cytokine production, stimulation of FcγRs with soluble IgG, as occurs under homeostatic conditions, results in inhibitory signaling that attenuates cytokine production (12, 13). Fourth, FcγR stimulation induces cell-intrinsic cytokine responses, thereby enabling cell-type and tissue-specific responses. For instance, while FcγR-TLR cross-talk enhances IL-10 production by DCs or macrophages, it attenuates IL-10 production by monocytes (7). In this review, we will summarize and discuss these four levels of regulation of FcγR-mediated cytokine production by human innate immune cells in the context of three immunological states: homeostasis, infection, and autoimmunity.

## Homeostasis: Inhibition of Cytokine Responses by FcγRs

**Figure 1:**
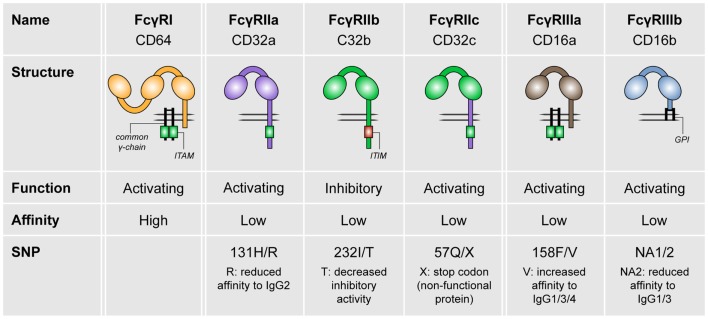
**The family of human FcγRs**. Human FcγRs are divided into three types: FcγRI, FcγRII, and FcγRIII. These receptors can be grouped by function (FcγRIIb is the only inhibitory receptor, whereas the other receptor are activating) or affinity to IgG (FcγRI is the only high-affinity receptor). For most of the FcγRs, SNPs are known that affect their affinity to IgG isotypes.

In humans, three different FcγR classes exist, which are FcγRI (CD64), FcγRII (CD32), and FcγRIII (CD16; see Figure 1). FcγRI is the only high-affinity receptor, indicating that it is able to bind monomeric IgG molecules. In contrast, all other FcγRs are low-affinity receptors and therefore require high-avidity binding by IgG immune complexes for appropriate binding and signaling (2–4). FcγRI, FcγRIIa, FcγRIIc (expressed only in a minority of individuals), FcγRIIIa, and FcγRIIIb are categorized as activating receptors, which mostly signal via so-called immunoreceptor tyrosine-based activation motifs (ITAMs). These ITAMs are situated either in their FcγR cytoplasmic tail (FcγRIIa, FcγRIIc) or in adaptor proteins such as the common γ-chain. In contrast, FcγRIIb is the only known inhibitory FcγR, which contains an immunoreceptor tyrosine-based inhibitory motif (ITIM) (3, 4).

Fc gamma receptors are widely expressed in virtually all hematopoietic cells, except for T cells. Most of these cells express both activating and inhibitory FcγRs, with the exception of NK cells (expressing solely FcγRIII) and B cells (expressing solely FcγRIIb) (3). Focusing on myeloid cells, monocytes express high levels of FcγRI and FcγRIIa, whereas FcγRIIb is moderately expressed and FcγRIII is expressed only on a subset of monocytes (4, 7). Both monocyte-derived DCs and DCs from blood express primarily FcγRIIa and FcγRIIb (4, 6–8). Macrophages express all classes of FcγRs, but particularly express high levels of FcγRIIa (4, 7, 14). In contrast, plasmacytoid DCs (pDCs) express FcγRs only at very low levels (4, 8). Here, we will first discuss the role of both inhibitory and activating FcγRs in homeostasis.

### FcγRIIb-mediated inhibition of cytokine production

Important for the control of cytokine production under homeostatic conditions is the balance between activating and inhibitory FcγRs. Studies in mice identified FcγRIIb as the main inhibitory receptor for various FcγR-mediated processes (3). ITIM-containing receptors, including FcγRIIb, perform their inhibitory functions via recruitment of phosphatases, specifically Src homology 2 (SH2) domain-containing phosphatase-1 (SHP-1) and SH2 domain-containing inositol phosphatase-1 (SHIP-1). These phosphatases are able to impede effector functions of ITAM-bearing receptors, including activating FcγRs, by interfering with activation of a variety of kinases and adaptor proteins (15, 16).

**Figure 2:**
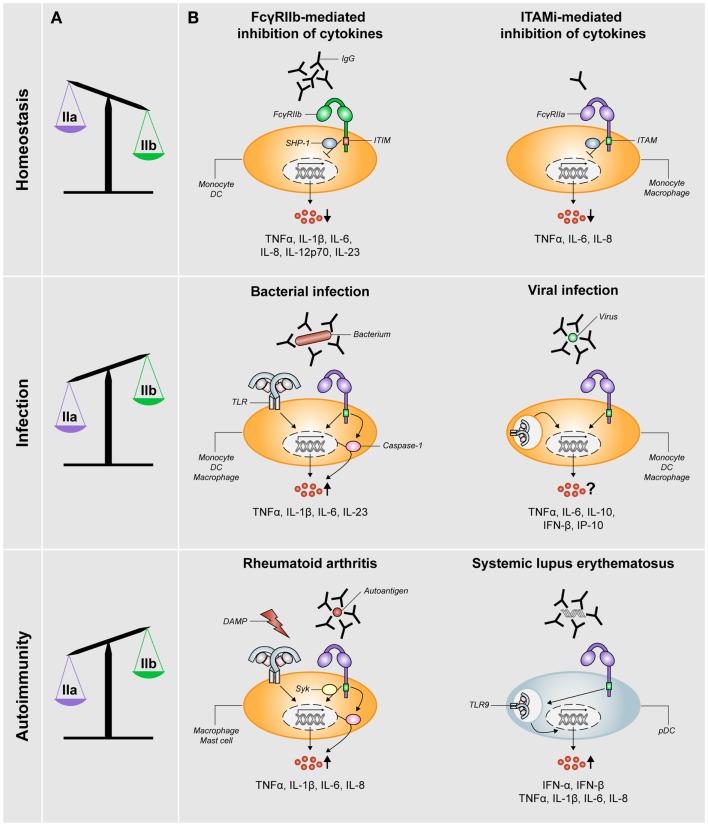
**FcγRIIa/b expression and FcγR-mediated control of cytokine production during homeostasis, infection, and autoimmunity**. **(A)** The balance of expression levels of activating FcγRIIa (IIa) and inhibitory FcγRIIb (IIb) is skewed toward FcγRIIb expression under homeostatic conditions, whereas it is skewed toward FcγRIIa under conditions of infection or autoimmunity (note: data on FcγR expression in RA patients are not fully consistent, see main text). **(B)** FcγRs orchestrate cytokine production under different conditions. *Homeostasis*: pro-inflammatory cytokine production is inhibited via either ITIM-mediated signaling via SHP-1 (or SHIP-1) downstream of FcγRIIb or ITAMi-mediated signaling downstream of activating FcγRs such as FcγRIIa. *Infection*: in the context of bacterial infections, specific pro-inflammatory cytokines are synergistically being upregulated (via upregulation of both transcription and caspase-1 activation) as a result of cross-talk between FcγRIIa and TLRs. For viral infections, the effect of simultaneous stimulation of FcγRs and TLRs recognizing viral structures on cytokine production is not yet clear. *Autoimmunity*: in RA, simultaneous stimulation of FcγRIIa (via IgG autoantibody-containing immune complexes) and TLRs (via disease-associated DAMPs) induces cross-talk similar to that upon bacterial infection, which results in synergistic upregulation of specific pro-inflammatory cytokines in a Syk-dependent manner. In the context of SLE, disease-associated immune complexes are being taken up by pDCs in an FcγRIIa-dependent manner and subsequently delivered to TLR9-containing lysosomes, which results in upregulation of production of type I interferons and pro-inflammatory cytokines.

The research on human FcγRIIb took a leap forward upon the development of a specific FcγRIIb-blocking antibody (8). Studies using human monocytes or DCs demonstrated that stimulation with IgG immune complexes, which simultaneously stimulate both activating and inhibitory FcγRs, hardly induce the production of any cytokines (6, 8, 17–19). In contrast, selective blockade of FcγRIIb under these conditions induces production of numerous cytokines and chemokines, including TNFα, IL-1β, IL-6, IL-8, IL-12p70, and IL-23 (8, 18, 20), which consequently promotes T-cell responses (8). These data demonstrate that the balance between activating and inhibitory FcγRs (schematically depicted in Figure 2A) effectuates a threshold for cell activation and consequent immune responses.

Regulation of this balance critically depends on the relative cell surface expression of activating and inhibitory FcγRs, which in turn is dictated by factors in the direct surroundings of the immune cells. Exposure to soluble, monomeric IgG, as occurs under homeostatic conditions, selectively reduces the expression of FcγRIIa on human DCs, thereby shifting the balance toward anti-inflammatory responses induced by inhibitory FcγRIIb (Figure 2A). In contrast, exposure to IFNγ, as occurs under inflammatory conditions, results in decreased FcγRIIb and increased FcγRI and FcγRIIa expression, thereby tilting the balance toward the induction of inflammatory cytokine production (8). Taken together, ITIM-containing FcγRIIb has a crucial role in regulating inflammatory responses under homeostatic conditions.

### ITAMi: Inhibitory signaling induced by circulating IgG

Although inhibitory signals were initially only associated with FcγRs that bear an ITIM (i.e., solely FcγRIIb) in the last decade, it has become clear that also ITAM-related receptors can negatively control inflammatory responses. This additional, anti-inflammatory function of ITAMs has been denoted inhibitory ITAM (ITAMi) (16, 21). It has recently been shown that circulating IgG monomers, as abundantly present in serum, induce ITAMi signaling by binding to human low-affinity receptors FcγRIIa (13) and FcγRIII (12). Besides inhibiting TLR-induced calcium responses, endocytosis, phagocytosis, and reactive oxygen species production, this ITAMi signaling also inhibits the production of pro-inflammatory cytokines and chemokines by human monocytes or macrophages, including TNFα, IL-6, and IL-8 (12, 13) (Figure 2B). Similar to ITIM-mediated inhibition, ITAMi-mediated inhibition requires SHP-1 recruitment (16). Subsequently, so-called inhibisomes are being formed, which are clusters containing FcγRs together with other receptors that are being inhibited, such as TLRs (12, 22). In addition, it has been described that small, soluble IgG complexes enhance TLR-induced anti-inflammatory IL-10 production by various subsets of human macrophages, whereas pro-inflammatory cytokines are not or hardly affected (23). However, it is yet unclear whether this elevated IL-10 production truly depends on ITAMi signaling.

In summary, FcγRs play an active role in the regulation of homeostasis, which is achieved by suppression of pro-inflammatory cytokine responses as well as promotion of IL-10 production via both inhibitory receptor FcγRIIb and ITAMi signaling of activating FcγRs.

## Infection: Induction of Pro-Inflammatory Cytokines by FcγRs

Whereas FcγRs suppress pro-inflammatory cytokines under homeostatic conditions, they are crucial in promoting inflammation upon infection. The ultimate immune response by myeloid cells induced in response to pathogens is not the result of stimulation of one single receptor, but rather is induced by cross-talk or collaboration between multiple receptors (24, 25). This collaboration between receptors induces an intricate regulation of cytokine production that effectuates pathogen- and tissue-specific immunity and prevents unbridled responses with detrimental effects.

Initially only PRRs, which recognize conserved microbial structures known as pathogen-associated molecular patterns (PAMPs), were considered to be able to induce innate response genes such as cytokines (26). However, recently it has become clear that also FcγRs play a major role in control of cytokine production. Due to the high levels of IgG directed against numerous antigens, invading pathogens are efficiently opsonized when they penetrate the body’s barriers, either directly during primary infection or at a later stage after generation of pathogen-specific IgG. Thus, in contrast to homeostatic conditions were IgG molecules are present in monomeric form, pathogen opsonization results in the formation of IgG complexes which can activate low-affinity receptors such as FcγRIIa. Consequently, innate immune cells sense these opsonized pathogens via PRRs and FcγRs simultaneously. Although FcγRs do not directly induce pro-inflammatory cytokines when stimulated individually (6, 8, 17–19), combined stimulation of FcγRs together with other receptors results in pathogen-specific cytokine responses. Below, we will discuss the role of FcγR-induced cytokine production in response to infection with different classes of pathogens.

### Bacterial infections

Immunoglobulin G seems to particularly play an important role in defense against bacteria, as patients with primary antibody deficiencies mainly suffer from bacterial infections, but hardly from fungal or viral infections (27). In healthy individuals, high amounts of cross-reactive IgG directed to bacterial antigens are present and consequently bacteria are efficiently being opsonized, even during primary infection (6). Importantly, FcγRII expression on monocytes and neutrophils is increased in patients with bacterial infections, compared to healthy controls or patients with viral infections (11). In addition, stimulation of TLR2 or TLR4, which predominantly recognize bacterial PAMPs, has been described to induce FcγRIIa expression on human monocytes (28). These findings indicate that exposure to bacteria makes myeloid cells more prone to recognize complexed IgG structures such as opsonized bacteria (Figure 2A).

Recent data show that cytokine induction by FcγRIIa plays an important role in directing antibacterial responses. IgG opsonization of various bacteria such as *Staphylococcus aureus* or *Klebsiella pneumoniae* strongly increases the production of particular pro-inflammatory cytokines by DCs, such as IL-1β, IL-6, IL-23, and TNFα, but not IL-12 (6). Induction of this specific cytokine profile skews T helper cell responses toward Th17, which is required for efficient eradication of extracellular pathogens and therefore appears to function as a natural mechanism to counteract bacterial infections. This synergistic cytokine response fully depends on cross-talk between FcγRIIa and TLRs, which are activated simultaneously on DCs upon encountering IgG opsonized bacteria. Mechanistically, FcγRIIa-TLR cross-talk in DCs is mediated by both enhancing the transcription of specific cytokine genes and via activation of caspase-1, which cleaves pro-IL-1β into its bioactive form (6). Besides DCs, FcγRIIa-TLR cross-talk also occurs in human monocytes and macrophages (7), indicating that this antibacterial mechanism is functional in multiple myeloid cell types (Figure 2B). In addition, several reports suggest that FcγRs and TLRs do not necessarily need to be activated simultaneously for this synergistic effect, since overnight activation of monocytes or DCs followed by stimulation with aggregated IgG still strongly increases TNFα production (10, 28, 29).

Monocytes exposed to IFNγ appear to have an additional, indirect mechanism of immune complex-dependent cytokine production. Upon LPS stimulation, exposure of these cells to immune complexes downregulates IL-10 receptor expression and inhibits IL-10 signaling in an FcγRI-dependent manner, which results in enhanced TNFα and IL-6 production (30). Importantly, this IL-10 loop was only observed in monocytes polarized in the presence of IFNγ, which induces FcγRI expression, but not upon M-CSF-induced differentiation (30), which stresses the importance of cytokines and differentiation factors in the micro-environment of immune cells for FcγR-mediated effects.

The importance of FcγRIIa in antibacterial responses is further emphasized by studies on the *FCGR2A* single nucleotide polymorphism (SNP) H131R. This SNP strongly affects binding affinity of the receptor to IgG2, the main isotype that is reactive to bacterial antigens (31). Multiple studies [reviewed by Van Sorge et al. (32)] indicate that *FCGR2A*-R131 homozygous individuals, carrying the receptor with low IgG2 affinity, are more susceptible to bacterial infections. However, this difference is unlikely to be caused by differences in FcγR-induced cytokine production, since FcγRIIa-TLR cross-talk is completely functional in DCs from *FCGR2A*-R131 homozygous individuals (7). Instead, this difference in susceptibility to bacterial infections may be explained by the finding that *FCGR2A-*R131 impairs IgG2-mediated phagocytosis (33). Apparently, while the low affinity for IgG2 of FcγRIIa-131R impairs uptake of opsonized bacteria, the binding of large IgG2 complexes is still sufficient to induce cytokines. This finding strengthens the idea that FcγRIIa-mediated cytokine production and uptake are regulated via distinct mechanisms.

The relevance of FcγR-mediated cytokine responses in combating bacteria is also indirectly indicated by the existence of immune escape mechanisms to evade this response. Interestingly, *Streptococcus pyogenes* produces Endoglycosidase S, an enzyme that is able to hydrolyze the heavy chain glycan of IgG molecules. As a result, the binding of IgG to FcγRIIa was strongly reduced (34), which impairs the antibacterial immune response. Additionally, *S. aureus* secretes a potent FcγRII antagonists, formyl peptide receptor-like 1 inhibitor (FLIPr) that competitively blocks IgG binding and subsequent IgG-mediated antibacterial effector functions (35).

Notably, FcγR-dependent control of cytokine production may not only depend on the presence of IgG. Also, members of the pentraxin family such as C-reactive protein (CRP) are known to interact with FcγRs. CRP is an acute-phase protein that is rapidly synthesized by the liver upon injury or infection and it is known to bind phosphocholine that is expressed on the surface of particular bacteria (36). It has been reported that CRP increases cytokine production, predominantly TNFα and IL-1β, by PBMC in response to *S. pneumoniae* via FcγRI and FcγRIIa (37).

In conclusion, FcγRs are critically involved in counteracting bacterial infections. Particularly, cross-talk between FcγRIIa and bacterial component recognizing TLRs in human myeloid cells selectively promotes the production of pro-inflammatory cytokines that play a crucial role in antibacterial immunity, such as TNFα and various Th17-promoting cytokines.

### Fungal infections

In contrast to bacterial infections, currently still little is known about the contribution of FcγRs to cytokine production in antifungal immune responses. However, it is known that opsonization of *Candida albicans* synergistically increases the production of TNFα by human monocytes or PBMC. This effect was largely dependent on extracellular signal-regulated kinases (ERK) (38). Fungi are recognized through multiple PRRs, including TLRs and C-type lectin receptors. Dectin-1 is one of the main cytokine-inducing C-type lectin receptors, which strongly contributes to antifungal immunity (26). However, FcγR co-stimulation with immobilized IgG does not enhance Dectin-1-induced TNFα production (7). This indicates that increased TNFα production upon exposure to opsonized *C. albicans* is likely to be dependent on cross-talk of FcγRs with TLRs, rather than with C-type lectin receptors. Furthermore, it strengthens the concept that FcγR stimulation does not simply enhance cytokine production induced by any given receptor, but instead specifically collaborates with particular (families of) receptors.

### Viral infections

The main focus of research on the role of antibodies and FcγRs during viral infections has been on virus neutralization, ADCC, antibody-dependent enhancement of infection, and phagocytosis, while data on FcγR-mediated cytokine responses in the context of viral infections is limited as well as conflicting. Recently, it has been shown that monocytes, DCs, and macrophages strongly upregulate TNFα and IL-6 production upon exposure to serum-opsonized Dengue virus, in an FcγRIIa-dependent manner (39). This is likely to be due to cross-talk of TLR3 and TLR7/8, recognizing virus-associated double-stranded or single-stranded RNA respectively, with FcγRIIa, as has been described recently (7). Others have confirmed upregulation of IL-6, as well as IL-10, upon serum-opsonized Dengue virus in a human monocytic cell line on both mRNA and protein level (40–42). In contrast, TNFα as well as IL-12 were reported to be downregulated in this assay (40).

Besides pro-inflammatory cytokines such as TNFα and IL-6, other cytokines and chemokines, particularly type I interferons and related chemokines, may be of great relevance in the context of viral infections (43). However, the effect of FcγRIIa (co-)ligation on these specific cytokines and chemokines has hardly been studied. The scarce data on this topic is not conclusive, as one study showed upregulation of IFNα and IFNβ by macrophages upon stimulation with opsonized Dengue virus (39) while others reported downregulation of IFNβ protein by a monocytic cell line upon Dengue opsonization (42). Interestingly, Posch and colleagues recently reported that exposure of DCs to opsonized HIV results in a decreased HIV-specific CD8^+^ T-cell response, in an FcγRIIa-dependent way. However, to which extent this effect was dependent on cytokines produced by DCs has not been studied (44). In conclusion, the role of FcγRs in cytokine production during viral infections (Figure 2B), and to what extend cytokine modulation is beneficial to the host or to the virus, is not yet clear.

### Parasitic infections

Recently, it was shown that erythrocytes infected with malaria-causing *Plasmodium falciparum* promote pro-inflammatory cytokines once opsonized with IgG. Particularly, opsonized infected erythrocytes, compared to unopsonized cells, induce high TNFα, IL-1β, and IL-6 production by human macrophages (45). Remarkably, the role of TNFα and other pro-inflammatory cytokines in parasitic infections, including *P. falciparum*, is ambiguous: TNFα has been identified to promote parasite killing, but it also contributes to development of severe malaria disease (46). Interestingly, the upregulation of pro-inflammatory cytokines was not transcriptionally regulated (45), in contrast to what was observed in DCs in response to opsonized bacteria (6). Induction of IL-1β upon exposure to opsonized infected erythrocytes was shown to be the result of FcγR-induced inflammasome activation (45), which is in agreement with previous studies using both DCs and M2 macrophages (6, 14). Although data on FcγR-mediated cytokines in antiparasitic responses is limited, it appears that similar to bacterial infections, IgG opsonization promotes specific pro-inflammatory cytokines upon parasitic infection.

## Autoimmunity: Undesired FcγR-Induced Cytokine Production

Although collaboration of FcγRs with other receptors to promote cytokine responses is beneficial in combating infections, undesired activation of this mechanism may contribute to the development of autoimmunity. RA, SLE, and several other autoimmune diseases are characterized by the presence of IgG autoantibodies and FcγR involvement in pathogenesis (47–49). In these diseases, IgG autoantibody-containing immune complexes can function as a danger signal that activates innate immune cells. We will here discuss the evidence of FcγR-mediated cytokine production in the context of several autoimmune diseases.

### Rheumatoid arthritis

RA is a chronic autoimmune disease occurring in 1% of the population and is characterized by inflammation and damage of the joints (50). Although the pathogenesis of RA is far from fully understood, it is clear that pro-inflammatory cytokines, predominantly TNFα, have a crucial role in the inflammatory process, as is emphasized by the great clinical improvement after neutralization of these cytokines (50). In recent years, the presence of autoantibodies, which is one of the hallmarks of RA, is beginning to be recognized as a contributing factor in inflammation and joint damage via the production of pro-inflammatory cytokines. The most prominent type of autoantibodies present in RA patients is anti-citrullinated protein antibodies (ACPA), which are present long before onset of disease symptoms and are mainly of the IgG isotype (49, 51, 52). Upon recognition of their antigen, e.g., citrullinated extracellular matrix proteins in the joint, autoantibodies form large, insoluble, and amorphous immune complexes (52) that enable their recognition by low-affinity FcγRs.

The importance of FcγRs in RA pathogenesis is indicated by various studies using mouse models for arthritis [reviewed by El Bannoudi et al. (49)], of which the use of human FcγRIIa transgenic mice may be the most relevant (53). These transgenic mice display a higher susceptibility to collagen-induced arthritis and developed more severe arthritis than wild-type mice (53, 54). Importantly, in a passive antibody transfer model, all FcγRIIa transgenic mice develop arthritis, while none of the control animals are affected. In addition, these transgenic mice spontaneously develop multi-organ autoimmunity (53).

In the context of RA, FcγR stimulation on myeloid cells has been shown to induce pro-inflammatory cytokines that are pivotal in RA pathogenesis, including TNFα, IL-1β, and IL-6. Precipitated or plate-bound IgG from serum or synovial fluid of RA patients, without any additional stimulus, induces TNFα production by healthy donor PBMC, predominantly monocytes, in an FcγRIIa-dependent manner (55–57). However, in these experiments, the resulting levels of TNFα were rather low (picogram-range), which indicates the marginal capacity of FcγRs to induce cytokine production when stimulated without any co-stimulation. Similar to their role in pathogen defense, FcγRs essentially need to collaborate with other families of receptors for the induction of physiological relevant cytokine responses. In RA synovia, this “second signal” most likely originates from the family of TLRs. Besides recognition of pathogens, TLR activation can occur through recognition of endogenous ligands, also referred to as damage-associated molecular patterns (DAMPs). These are abundantly present in RA synovia as a result of tissue damage and cell death (58). Indeed, activation of macrophages with IgG immune complexes-containing citrullinated fibrinogen, which activates both FcγRIIa and TLR4, strongly induces the production of TNFα (59–61) (Figure 2B). A similar effect of FcγRIIa-TLR cross-talk has been observed in human mast cells, which results in synergistic upregulation of IL-8 production (62).

In addition, FcγR-TLR cross-talk may promote inflammation in RA patients by interfering with the immunosuppressive function of M2 macrophages. Although macrophages are a heterogeneous population of cells that can differentiate into a full spectrum of different phenotypes, macrophages are generally being categorized into either M1 macrophages, which are classically activated macrophages with pro-inflammatory properties, or M2 macrophages, which display anti-inflammatory, regulatory, and/or wound healing properties. Importantly, while M2 macrophages are known to suppress inflammation in disorders such as tumor formation, atherosclerosis, and obesity (63), in RA patients FcγR-TLR cross-talk converts M2 macrophages to promote inflammation (14). While the general phenotype of M2 macrophages is retained, stimulation of these cells with IgG immune complexes and TLR ligands induces the selective induction of RA-associated cytokines TNFα, IL-1β, and IL-6, and promotes Th17 responses, in a spleen tyrosine kinase (Syk)-dependent way (14). Since the conventional function of M2 macrophages, i.e., preventing disproportionate immune activation and mediating tissue repair, is abrogated, this may thereby contribute to excessive inflammation as observed in RA patients.

Considered the importance of the balance of activating versus inhibitory FcγRs in controlling inflammation, numerous studies have investigated FcγR expression levels on immune cells of RA patients. However, the data on this are far from consistent. Some studies found no differences between RA patients and healthy controls (60, 64–66), whereas others reported that monocytes, mo-DCs, monocyte-derived macrophages, and synovial macrophages of RA patients displayed elevated levels of activating receptors FcγRIIa and FcγRIII, while expression of inhibitory receptor FcγRIIb was similar to healthy controls (9, 10, 29, 57, 67, 68) (Figure 2A). The reasons for these inconsistent findings are still unclear, but may involve differences in the stage of disease, donor variation, and disease heterogeneity.

Taken together, the body of evidence for a causative role of FcγRs, predominantly in synergy with TLRs, in the induction of inflammation in RA is increasing. As such, these data support the concept that the occurrence of IgG autoantibodies is not merely an epiphenomenon, but in fact actively contributes to RA pathogenesis.

### Systemic lupus erythematosus

Another well-known IgG immune complex associated autoimmune disease is SLE. SLE is a chronic, systemic autoimmune disease that can affect virtually any organ, but primarily kidneys, skin, lungs, brain, and heart. SLE is characterized by autoantibodies to DNA, RNA, and other nuclear structures. The key cytokine in the inflammatory process in SLE is considered to be IFNα, which is mainly produced by pDCs (48, 69). A meta-analysis covering 17 studies revealed that *FCGR2A*-R131 is a significant risk factor for SLE (70), suggesting FcγRIIa is involved in SLE pathogenesis. Furthermore, it is known that ligation of TLR7 and 9, which are endosomal receptors that recognize RNA and DNA structures and are constitutively expressed by pDCs, induces IFNα production. The localization of TLR7 and 9 in the endosomal compartment ensures that under physiological conditions, these TLRs are shielded from self-RNA or self-DNA at the exterior of cells, for example from dying cells (69).

FcγRIIa has been shown to be important in inducing IFNα in SLE patients, via cooperation with TLRs. Means and colleagues elegantly showed that FcγRIIa facilitates uptake of DNA-containing immune complexes and delivery to intracellular lysosomes comprising TLR9 in human pDCs (71). This FcγRIIa-dependent activation of TLR9 results in production of IFNα, as well as other cytokines and chemokines such as TNFα, IL-1β, IL-6, and IL-8 (71, 72) (Figure 2B). Interestingly, this mechanism of FcγRIIa-induced upregulation of cytokines in pDCs differs from that in other cell types. While in pDCs the amplification of TLR-induced cytokine production critically depends on FcγRIIa-dependent uptake of immune complexes (71), cytokine production by FcγRIIa in DCs and macrophages is independent of uptake (6, 14). Thus, although via a different mechanism and in a different cell-type than in RA, FcγRIIa also contributes to the pathogenesis of SLE via amplification of cytokine production.

### Systemic sclerosis

Systemic sclerosis (SSc) or scleroderma is a heterogeneous autoimmune connective tissue disease of unknown etiology, which is characterized by excessive fibrosis in the skin and internal organs, vasculopathy, and immune abnormalities. Autoantibodies are present in more than 95% of SSc patients, which are directed against a variety of nuclear, cytoplasmic, and extracellular autoantigens (73). In addition, SSc is characterized by the release of endogenous TLR ligands, which form immune complexes by the binding of autoantibodies (74). Reminiscent of what has been observed in pDCs for SLE immune complexes (71), stimulation with SSc immune complexes induces IFNα production by PBMC, which is dependent on FcγRII-mediated uptake of immune complexes and the presence of RNA, suggesting involvement of TLR7 (75). Similar to SLE, this mechanism may contribute to the IFN type gene “signature” as observed in many SSc patients (76).

### Other autoimmune diseases

Besides RA, SLE, and SSc, FcγR-dependent modulation of cytokine production may play a role in the pathogenesis of several other disorders characterized by IgG autoantibodies. In principle, FcγR-TLR cross-talk can be induced in any disorder involving immune complexes, endogenous TLR ligands, and FcγR- and TLR-expressing immune cells. Although there is little direct evidence, several diseases are likely to fulfill these criteria, including Sjögren’s syndrome, pemphigus, and multiple sclerosis (77–80). Future studies are required to elucidate whether and to what extent FcγR-mediated cytokine production indeed is involved in these autoimmune diseases.

## Modulation of FcγR-Induced Cytokine Production: Opportunities for Therapeutic Intervention

We have discussed that stimulation of FcγRs with IgG immune complexes, predominantly in cooperation with PRRs such as TLRs, promote inflammatory cytokine responses. On one hand, this is beneficial to the host, since it allows us to efficiently counteract infections with bacteria and possibly also other classes of pathogens. On the other hand, activation of this mechanism can also have detrimental effects, since it may promote inflammatory responses leading to autoimmunity. Therefore, modulation of FcγR-induced cytokine production in the context of infection and autoimmunity may provide opportunities for therapeutic intervention, either by reducing or by enhancing these inflammatory responses.

An important example of FcγR-related therapy is the use of intravenous immunoglobulin (IVIG). IVIG was initially used as an IgG replacement therapy for immunocompromised patients, but paradoxically also has general anti-inflammatory effects. Our understanding of the anti-inflammatory effect of IVIG is still far from complete and is beyond the scope of this review. Excellent reviews by others (81, 82) summarize several modes of action of IVIG in autoimmunity, including blockade of interaction of immune complexes with activating low-affinity FcγRs. Moreover, IVIG administration modulates the balance between activating and inhibitory FcγRs, predominantly as a result of increased expression of inhibitory receptor FcγRIIb (81, 82). In addition, recently it has been described that IVIG preparations contain anti-FcγRII and anti-FcγRIII antibodies (83), which may interfere with binding of these FcγRs to disease-associated autoantibody structures.

An alternative approach to interfere with FcγR-mediated inflammation is to specifically provide IgG molecules that preferentially bind to and activate the inhibitory receptor FcγRIIb. Indeed, recently an anti-CD19 antibody carrying an Fc region with over 400 times greater affinity to FcγRIIb compared to FγRIIa has been engineered (84). Co-engaging of the B-cell receptor complex together with FcγRIIb by this engineered antibody suppressed B-cell activation and function, including IgG secretion and IL-6 production (85–87), which therefore may be of therapeutic benefit in IgG-mediated autoimmune diseases such as RA and SLE.

Moreover, it may be promising to specifically interfere with downstream molecules involved in FcγR-modulated cytokine production. Although the mechanism of FcγR-modulated cytokine production is still largely unidentified, Syk is known to be pivotal for cytokine production induced by FcγRIIa-TLR cross-talk (14). Interestingly, therapeutic inhibition of Syk using oral small molecule inhibitor R788 indeed significantly reduces disease activity in RA patients (88). Although Syk is probably also required for other immunological processes, these trials illustrate the potential therapeutic possibilities of interfering with FcγR-induced cytokine production. Similarly, identification of other key signaling molecules of FcγR cross-talk in the future may give rise to additional targets for therapy that could be blocked using small molecule inhibitors.

While inhibition of FcγR-induced cytokine production may be beneficial to attenuate inflammation in autoimmunity, enhancing inflammation may be useful in the context of bacterial infections or solid tumors. Considered its ability to amplify the induction of Th17 responses by antigen-presenting cells such as DCs, an adjuvant that would simultaneously cross-link activating low-affinity FcγRs and TLRs may function as a powerful new vaccination strategy for establishing effective antibacterial memory responses. In addition, during both chronic bacterial infections and solid tumors, the local environment is dominated by the presence of anti-inflammatory or suppressive M2 macrophages, which attenuate the generation of effective antibacterial or antitumor immune responses (63, 89, 90). As FcγR-TLR cross-talk is known to elicit pro-inflammatory cytokine responses by M2 macrophages (14), local induction of this response may greatly enhance the induction of antitumor immunity. Since IgG antibody therapy is already used for the treatment of solid tumors (91), the coupling of TLR agonists to these IgG antibodies for the local induction of inflammation may be a very useful tool to further enhance the efficacy of current treatments.

Taken together, additional knowledge on the specific cytokine profile induced by different cell types and the identification of the underlying molecular mechanisms are promising subjects for future research, since this may lead to novel therapeutic strategies for a large variety of disorders, including chronic (bacterial) infections, tumor formation, and IgG autoantibody-associated autoimmune diseases.

## Concluding Remarks

Here, we have described and discussed a novel function of human FcγRs in shaping cytokine responses and thereby orchestrating context-dependent immunity. Although the cytokine profile induced by stimulation of activating FcγRs varies depending on the cell-type and the combination of stimuli, it appears that TNFα is the key cytokine that is being upregulated in almost all cell types in response to FcγR co-stimulation. An important remaining question is the identity of the (classes of) receptors are able collaborate with FcγRs for the amplification (or inhibition) of cytokine production. Thus far, FcγRs have been shown to synergize with TLRs, IFNγ receptor, and IL-1 receptor, but not with various other cytokine receptors or C-type lectins (7). Alternatively, besides FcγRs also other classes of Fc receptors, including FcεR and FcαR, may affect cytokine production upon collaboration with PRRs or other receptors (92). Further identification of collaborating and non-collaborating receptors will offer new insights into the shaping of cytokine responses by myeloid cells and thereby may provide new perspectives for future therapies.

While in this review we have completely focused on the cytokine shaping properties of FcγRs, it is important to realize that FcγRs are responsible for many other processes, including uptake, antigen presentation, and ADCC. Therefore, in view of vaccination or other therapeutic strategies, it would be useful to specifically interfere with one aspect of FcγR-mediated effects, while leaving other functions intact. In this regard, the recent findings that indicate that FcγR-mediated phagocytosis and cytokine production are regulated via distinct mechanisms may provide valuable clues. However, to fully exploit these differences, more knowledge about mechanistic properties of these different FcγR functions in the human immune system is required.

## Conflict of Interest Statement

The authors declare that the research was conducted in the absence of any commercial or financial relationships that could be construed as a potential conflict of interest.

## Abbreviations

ACPA, anti-citrullinated protein antibodies; ADCC, antibody-dependent cellular cytotoxicity; CRP, C-reactive protein; DAMP, damage-associated molecular pattern; DC, dendritic cell; FcγR, Fc gamma receptor; IgG, immunoglobulin G; ITAM, immunoreceptor tyrosine-based activation motif; ITAMi, inhibitory immunoreceptor tyrosine-based activation motif; ITIM, immunoreceptor tyrosine-based inhibitory motif; IVIG, intravenous immunoglobulin; PAMP, pathogen-associated molecular pattern; pDC, plasmacytoid dendritic cell; PRR, pattern recognition receptor; RA, rheumatoid arthritis; SH(I)P-1, Src homology 2 domain-containing (inositol) phosphatase-1; SLE, systemic lupus erythematosus; SNP, single nucleotide polymorphism; SSc, systemic sclerosis; Syk, spleen tyrosine kinase; TLR, toll-like receptor.
